# Fermentation of Agri-Food Waste: A Promising Route for the Production of Aroma Compounds

**DOI:** 10.3390/foods10040707

**Published:** 2021-03-26

**Authors:** Jasmine Hadj Saadoun, Gaia Bertani, Alessia Levante, Fabio Vezzosi, Annalisa Ricci, Valentina Bernini, Camilla Lazzi

**Affiliations:** Department of Food and Drug, University of Parma, Parco Area delle Scienze 49/A, 43124 Parma, Italy; jasmine.hadjsaadoun@unipr.it (J.H.S.); gaia.bertani@unipr.it (G.B.); alessia.levante@unipr.it (A.L.); fabio.vezzosi.fv@gmail.com (F.V.); annalisa.ricci@unipr.it (A.R.); camilla.lazzi@unipr.it (C.L.)

**Keywords:** waste, valorization, fermentation, lactic acid bacteria, flavor

## Abstract

Food waste and byproducts are generated along the entire food processing and storage chain. The large amount of waste deriving from the whole process represents not only a great economic loss but also an important ethical and environmental issue in terms of failure to recycle potentially reusable materials. New, clear strategies are needed to limit the amount of waste produced and, at the same time, promote its enhancement for further conversion and application to different industrial fields. This review gives an overview of the biological approaches used so far to exploit agri-food wastes and byproducts. The application of solid-state fermentation by different microorganisms (fungi, yeasts, bacteria) to produce several value-added products was analyzed, focusing on the exploitation of lactic acid bacteria as workhorses for the production of flavoring compounds.

## 1. Are We Sure They Are Really Waste?

In recent decades, a new challenge concerning the reduction of food waste (FW) and food loss has been raised for the world population. Indeed, food production is increasing annually worldwide, and the Food and Agriculture Organization of the United Nations (FAO) has estimated that one-third of the products intended for human consumption (corresponding approximately to 1.3 billion tonnes/year) is wasted or lost every year [1].

This event, in developing countries, mainly originates from the first steps of the food supply chain, due to technical and management limits in the post-harvest, processing, and distribution steps. On the other hand, in medium- and high-income countries, food is lost or discarded in the final steps, by retailers and consumers, due to the high productivity and quality standards required by the market [2]. As a result, many resources are lost in terms of land, water, and nutrients, and therefore the production, processing, manufacturing, and transport steps become a waste of energy. 

Considering the growth of the global population projected to 2050, to ensure an appropriate food supply, food production must increase in the next decades [3]. This will cause a rise in FW, ultimately posing a serious problem in terms of waste management and disposal.

Thus, the modern challenge is to minimize FW, optimize production processes, and move from a linear consumption and production model to a new one organized according to a circular economic strategy. In this view, FW is considered as a byproduct and resource, and several studies about waste and byproduct valorization have been conducted in recent decades [4,5,6].

FW is defined as any part of food that is discarded, regardless of its potential content of compounds retaining a high value [7]. Depending on its origin or production, FW can be characterized by a variable chemical composition of carbohydrates, proteins, lipids, and other components that could be used in different industries and fields [8], such as biorefineries, biomaterials, pharmacy, cosmetic, and aroma industries. Using food byproducts could be advantageous for the aroma industry due to market demand for natural products and their low material cost. 

The aim of this review is to provide insights into biotechnological strategies performed to exploit waste and byproducts from the agri-food chain. In particular, this work is focused on the application of solid-state fermentation (SSF) to produce several value-added products such as flavor and aroma compounds. To better delineate a specific topic, considering the metabolic potential of lactic acid bacteria (LAB), we decided to present a detailed overview of their employment in the production of natural flavors. Indeed, the involvement of these microorganisms “generally recognized as safe” (GRAS) in food processing and their role in flavor formation is well recognized, but their potential for the bioconversion of wastes and byproducts has only recently been considered. In this review, the literature is critically examined, giving an overview of the food waste management issue and presenting fermentation as an opportunity for FW bioconversion into new value.

## 2. Agri-Food Waste: A Rising Problem or a Valuable Resource?

Among the different food sectors, it is estimated that fruit and vegetables represent a large part of waste production, notably in the detail that about 45% of the total produced amount is lost in the production and consumption chains, generating a great quantity of waste material [9,10]. Wastes and byproducts can be classified into four source groups, according to the step of the agri-food chain in which they are generated: (i) in the fields, before harvesting, due to pest infestation and crops damaged by unfavorable weather conditions; (ii) in post-harvest and transport, where spoiled and bruised fruit and vegetables are discarded; (iii) in the different manufacturing steps process such as peeling, washing and slicing; (iv) in retail and the markets, due to natural spoilage at the end of shelf life [11].

The recovery and management of these wastes are not trivial. Seasonality, distribution across a territory, and perishability due to the high content of water and nutrients and the heterogeneity of the products may represent possible difficulties and problems for agri-food waste management [12]. 

A feasible and economically sustainable agri-food waste recycling program requires large volumes of raw materials concentrated in the same area, a high degree of homogeneity, and a careful analysis of downstream costs [13]. In keeping with this, industrial symbiosis could be a productive and useful strategy. The FWs generated by diverse companies could be transferred to other industries, which could transform them for other purposes in a circular economic model [14]. 

In recent decades, the fate of organic waste was different in different types of areas. In rural areas and farms, organic wastes were commonly used as livestock feed or were composted into humic substances used as fertilizers in fields. In urban areas, household wastes with a more complex composition were destined for incineration or landfilling, thus posing significant environmental problems with respect to air pollution and groundwater contamination [15]. 

The theory of waste valorization is strictly associated with sustainable technologies for recycling and reuse. The concept behind waste valorization is to enhance the value of a product by converting waste into other resources providing an added value. The resulting products could include new chemicals, materials, fuels, and energy, just like a lot of other products advantageous to local and global economies.

Furthermore, the valorization and recycling potential of agri-food wastes and byproducts can ensure sustainable food production and at the same time guarantee food security. Interestingly, some materials derived from the food industry can be reused thanks to their distinctive properties, exploitation of their physic-chemical characteristics can occur in many different industrial sectors. Lignocellulosic byproducts like soy and corn stalks or wheat straw could be used in the paper industry or as reinforcement in biodegradable polymer matrices to prepare building products with high strength [16]. 

With this in mind, currently, the most promising frontier seems to be biorefineries. Biorefineries are industries that start from biomass feedstock and, through extraction processes or chemical and biological reactions, can recover the nutrients to create value-added products and green energy [17]. 

In these new industrial realities, organic materials can be treated by various techniques such as SSF, submerged fermentation (SmF), or anaerobic digestion (AD). For example, chemical compounds like bioethanol and biobutanol are obtained from starchy and lignocellulosic byproducts through fermentation by *Saccharomyces cerevisiae* and *Clostridium acetobutyricum*, respectively. In this case, saccharides must be enzymatically pre-treated to break down the polymer chains into glucose monomers which are subsequently metabolized by microorganisms [12]. Different types of biofuels can be obtained depending on their diverse starting substrates. For example, biodiesel is obtained from the transesterification of vegetable oils and animal fats, whereas methane and biohydrogen are both produced both the AD of any biodegradable substrate and by pyrolysis [17]. Another source of byproducts for bioethanol production is the sugarcane industry, whose byproducts have been proposed for AD, microbial fermentation, and microalgae cultivation [18].

With production of over 700 Mt/year, rice, wheat, and corn crops represent one of the main food sources worldwide. After their processing and milling, huge volumes of byproducts like stalks, straws, and husks remain [19], which could be used for biofuel production due to high cellulose and hemicellulose content. 

Being biodegradable and compostable, food and beverage companies have recently begun paying great attention to biomaterials coming from renewable sources, from the perspective of environmental protection. Those are only a few examples related to new materials obtained from agri-food wastes. Bioplastic materials can be produced through the lactic acid fermentation of agro-industrial residue and household waste through engineered microorganisms. Polylactate (PLA), deriving from the polymerization of lactic acid monomers, is mainly used for the manufacturing of compostable products with a short shelf-life such as films and bags for packaging, thanks to its good transparency, biocompatibility, and processability [20]. Similarly, polyhydroxyalkanoates (PHAs) with the most representative being poly[(R)-3-hydroxybutyrate] (PHB), are used in different applications, including packaging [21].

Many compounds are also extracted and used as additives, colorants, or ingredients in food. Among them are pectin from citrus and passion fruit peels, apples, and peach pomace, which is used as a gelling agent, thickener, and food stabilizer [22]; carotenoids from tomato skins and pomace (mainly lycopene and β-carotene), carrot byproducts, mango, and orange peel. Dietary fiber can be recovered from banana peels and used in baking [8]. Orange juice fiber byproducts have been highlighted for potential use as a fat replacement in ice cream [23]; and finally, anthocyanins can be extracted from berry byproducts, grape pomace, and several exotic fruits [24]. In particular, bioactive compounds like polyphenols, flavonoids, and minerals are extracted from the losses and byproducts of fruit and vegetable industries and find use in the production of the functional food and nutraceutical sectors [25].

This policy of recycling and reuse requires not only technical knowledge but also a change of the global mindset. Wastes and by-products are often seen as useless stuff to throw away, with no thought given to their possible reuse. Furthermore, it is necessary that these wastes be seen from a perspective wider than just one company or sector. To reach the waste recycling goal, it is important that industries communicate with each other to establish a close system of valorization where an industrial symbiosis between diverse sectors can be a productive and useful strategy.

## 3. Repurposing Agri-Food Waste by Solid-State Fermentation for the Production of Aroma Compounds

Fermentation is a well-known technique dating back to ancient civilizations for the production of food commodities such as bread, wine, and fermented milk. Microbial transformation made the products particularly appreciated for their easier digestibility, flavor, and longer shelf life. Nowadays, there is great interest in improving the health, nutritional, technological, and organoleptic qualities of fermented foods, and, thanks to the development of new starter cultures, it is possible to guide microorganisms to different substrates for the production of new compounds, including flavors [26]. The biodiversity of microorganisms can be exploited not only to produce foods with peculiar and appreciated aromatic notes, but also to convert diverse precursors into fine biochemicals such as aroma compounds and fragrances, through biocatalysis.

Fermentation is a process operated by microorganisms to break down organic compounds to obtain energy through anaerobic metabolism. This biological process is characterized by low costs, low energy consumption, and low wastewater generation, and it can be exploited to repurpose organic wastes into value-added products [27]. In SSF, fungi, yeasts, and bacteria grow on the surface of various organic substances, which act as physical support for their development without adding water [28]. Fungi and yeasts are the microorganisms of choice for this application, which is conducted at a moisture content between 40–80% [29]. Alternatively, SmF can be applied with a moisture content of about 80–95%.

Each technique has both advantages and limitations: SmF is routinely applied to the production of a variety of products ranging from beverages such as wine and beer to more sophisticated methods such as the cultivation of animal and plant cell cultures for biomedical applications. Despite its broad range of applications, SmF has some drawbacks regarding process scale-up, due to the requirement of large volumes of water compared to low yield [30]. SSF, on the other hand, is traditionally applied in the manufacture of various Asian fermented foods, and has recently gained attention due to its low operating cost, reduced water consumption, and the lack of requirement for sophisticated bioreactors. However, the drawbacks of this technique are the limited control of the environment within the bioreactor and the high costs for end-product recovery and downstream processing [31]. The choice of the most suitable media and microorganisms for the optimization and planning of the downstream processing steps, as well as the possibility of using low- to zero-cost substrates such as food waste, represent the keys to the success of this technique [32].

As described in different studies, several added-value products can be recovered from agri-food waste substrates after fermentation, such as antibiotics, pigments, biosurfactants, hydrolytic enzymes, plastics, pesticides, and bioactive compounds [28,33,34,35,36]. Several agro-industrial wastes can be used as immobilization carriers in SSF, as reported by Orzua et al. [37], and represent an opportunity for the synthesis of industrially relevant metabolites.

Aroma compound production is a promising field for the application of SSF. Flavor compounds can be chemically synthesized, extracted directly from a natural matrix, or derived from biotechnological processes [38]. These approaches offer the possibility of obtaining additives suitable for various industrial applications, which can be labeled as “natural” and environmentally friendly as they require fewer solvents for extraction, compared to chemical synthesis methods [39]. The flavors obtained by these biotechnological processes can find applications not only as food ingredients, but also in the chemical, pharmaceutical, and cosmetic industries with the purpose of enhancing or modifying the original aroma of a product. In this way, they also acquire great importance over the acceptance of products by the consumer market [40].

**Figure 1:**
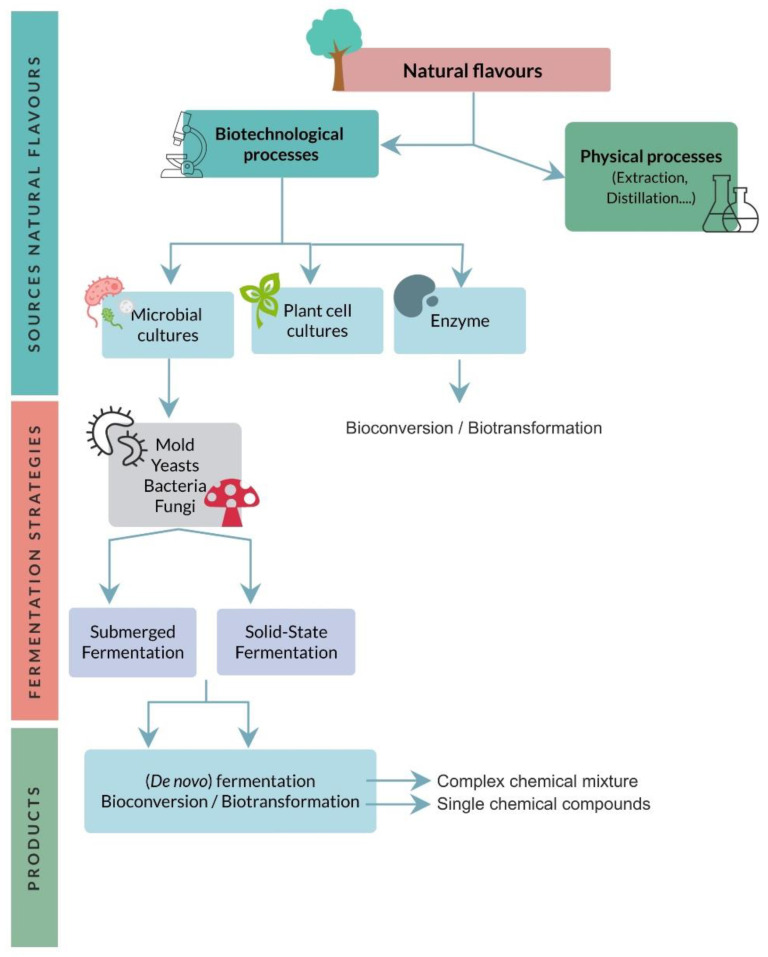
Processes to obtain natural flavors.

Currently available biotechnological processes for aroma production (Figure 1) make use of enzymes, microbial cultures, or, less frequently, plant cell cultures [41].

The application of enzymes for the production of aroma compounds is based on their addition to the substrate during the flavor production process. Enzymes like lipase, protease, glucosidase, and cellulase can act on specific precursors of aromatic compounds. The result is the bioconversion of organic material into an aromatic product through a single- or multi-step catalyzed reaction [30]. One of the most promising applications is the use of lipolytic enzymes in order to produce esters [42], even if the scaling-up of this application to an industrial scale remains complicated due to the high cost. The pre-treatment of grease waste with lipolytic enzymes prior to SSF was also proposed as an effective strategy to recover fatty acids from degraded grease waste [43].

When we describe the effect of a microbial culture on a substrate, we can distinguish between bioconversion/biotransformation and fermentation. In biotransformation, the microorganism converts a precursor into a product of interest through a single- or multi-step reaction such as the conversion of ferulic acid into vanillin and the stereo- or regio-selective changes of terpenes, as reviewed by Sales et al. [38]. The biotransformation process is particularly promising for the use of engineered microorganisms. Starting from the enzyme which catalyzes the reaction, and knowing how the enzyme works, it is possible to insert the gene coding for that enzyme into the genomes of high-producing and better adaptable microorganisms to increase the effectiveness and efficiency of the process. The gene coding for the lipoxygenase of *Pleurotus sapidus* has been cloned, for example, in *Escherichia coli*, thus enabling it to convert valencene into the grapefruit flavor nootkatone [44].

Cereal bran and other agricultural wastes such as sugar beet pulp, rice bran oil, palm oil biomass, pineapple byproducts have been studied as sources of ferulic acid, a precursor for the conversion of bio-vanillin with natural or engineered bacteria [45,46]. 

Biotransformation is an easier process to apply on an industrial scale since it leads to the production of single-aroma compounds, but it is difficult to carry out using waste [47]. Furthermore, the use of genetically modified organisms is not perceived as truly “natural” by consumers [48].

In fermentation (de novo synthesis), an entire metabolic pathway is involved. The catabolism of carbohydrates, proteins, and lipids contributes to the production of the primary metabolites, which are subsequently converted into a mixture of aromatic compounds [38]. 

When agri-food wastes or byproducts are used as a substrate, glucose supplementation is often required to support the initial growth of microorganisms, although high concentrations might lead to catabolite repression phenomena [49]. An interesting strategy to overcome nutrient limitations occurring in SSF on agri-food waste is to mix different waste substrates, thus developing fermentation substrates that do not require nutrient supplementation. With this aim, an SSF of mixed agri-food wastes with *Kluyveromyces marxianus, S. cerevisiae*, or an undefined mixed culture from kefir, has been demonstrated as being a promising approach for the development of biorefineries aimed at the production of biomasses and volatile aroma compounds [50].

The production of specific compounds has been demonstrated to be inducible by adding precursors. For example, the addition of leucine and valine to growth substrates including agri-food waste, leads to the formation of isoamyl acetate with a strong banana aroma [51,52], due to the Ehrlich pathway that leads to the catabolism of the amino acid and the production of esters as final products [53]. Other derivatives of the Ehrlich pathways are the rose-scented volatile compounds 2-phenylethanol and 2-phenethyl acetate, which were synthetized through the SSF of sugarcane bagasse upon the addition of L-phenylalanine as a precursor, from the yeasts *K. marxianus* and *Pichia kudriavzevii* [54,55]. 

Several studies have been conducted on the use of microbial cultures (especially molds and yeasts) growing on agri-food wastes and byproducts, to produce aromatic compounds, and a list of the main results is reported in Table 1. 

The optimization of SSF approaches relies consistently on the isolation of novel strain/substrate combinations, and strain selection a key point of the process due to the strain-specific capabilities of volatile compound production [66]. It is also known that the microbial growth phase can affect the production of volatile compounds [70,71], and the optimization of SSF processes suggests that the metabolic state of microbial cells can influence their synthesis [72]. Yet, the limited knowledge of bacterial physiology during SSF and the regulation of the pathways involved in aroma formation represents a limit to the optimization of strain selection and operating procedures [73].

The fermentation processes are followed by bioseparation processes such as extraction, purification, and the recovery of the compound of interest. Due to compound volatility and low solubility, recovery is the most difficult step, particularly for flavor components [74]. Deep knowledge of the properties of the target compounds and the matrix in which they are dispersed is necessary, to choose the most appropriate extraction method, to increase selectivity and efficiency, and thus to obtain the maximum recovery of the product from SSF. 

**Figure 2:**
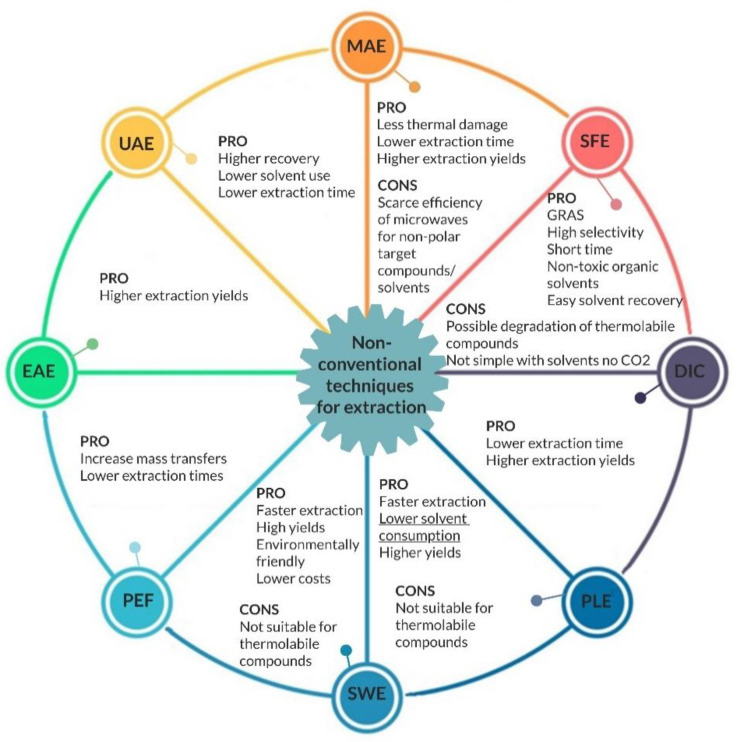
Operating principles of nonconventional techniques with their main advantages (PRO) and disadvantages (CONS) compared to traditional techniques. UAE, ultrasound-assisted extraction; MAE, microwave-assisted extraction; SFE, supercritical fluid extraction; DIC, instant controlled pressure drop-assisted extraction; PLE, pressurized liquid extraction; SWE, subcritical water extraction; PEF, pulsed electric field; EAE, enzyme-assisted extraction.

There are many techniques available for the isolation of flavor compounds, and although there is no correct technique in general, the challenge is to find the one most suitable capable of extracting the desired flavors in the best way, avoiding losses of volatile compounds during the process due to the aeration of the SSF bioreactors [75]. A combination of different extraction and separation techniques (hybrid processes) often proves beneficial for large-scale applications [76]. Next to the traditional extraction methods, the development of non-conventional techniques aims to improve the efficiency of extractive processes of bioactive compounds. These techniques can reduce the time and temperature of extraction while maintaining high selectivity and high yield using less dangerous solvents, and that is why they are considered “green techniques” [77]. Considering all these aspects, it would be desirable that an increasing number of studies report preliminary cost analyses, to provide orientation for future development of this industrial process [50]. Figure 2 summarizes these techniques, highlighting the pros and cons of each. 

## 4. Lactic Acid Bacteria: Biological Resources for Volatile Compound Production

LAB are widely used in the food industry as starters, to drive fermentation processes, and as probiotic sources. LAB are easy to employ because they are recognized as GRAS, have a simple metabolism, can grow on many different carbon sources, and have a good tolerance to environmental stresses such as pH and temperature [78]. In recent years, the development of genome sequencing techniques and genetic tools has widened the range of applications, helping to control the bioproduction of value-added products and standardize the process. All of these characteristics make it possible to exploit LAB in biorefineries for the production of various types of value-added products, such as lactic acid, plastic polymers, ethanol, exopolysaccharides (with thickener and prebiotics function), antimicrobial molecules, food aromas, and sweeteners (sorbitol, mannitol, l-alanine) [78]. 

Recently, various studies have been dedicated to the lactic acid fermentation of different substrates [79,80] using *Lacticaseibacillus rhamnosus* for the evaluation of the aromatic component which undergoes modifications during the process. Fermentation can be used to increase aromatic notes [81] or to reduce off-flavor components in the products [82]. Changes to the aromatic profile derive from the metabolism of bacteria which, depending on the various nutritional compounds available, produce different metabolites. Microorganisms metabolize carbon sources for growth and energy production during fermentation. The resulting metabolites can be both aromatic compounds and aroma precursors [53]. These compounds have specific sensory attributes, for example, esters are characterized for the most part by sweet smell [30], while aldehydes usually bring floral or fruity notes [83]. 

Important and complex aromatic molecules are known to be generated by LAB during the fermentation of dairy foods. These compounds are synthetized as a result of the primary metabolism of carbon sources (lactic acid and mixed acid fermentation), or by proteolisys and other secondary metabolisms that occur during the cheese ripening step. The buttery flavor seems to be the most interesting compound produced by LAB in qualitative and quantitative terms and is widely used in bakery. The molecule that best expresses this flavor is diacetyl, followed by acetoin, butanediol, and acetaldehyde. The above, and other metabolites such as ethanol and acetate, derives from pyruvate, which gives a typical flavor to fermented foods. Only some species of LAB, with the ability to metabolize citrate, can produce diacetyl [84]. The microbial synthesis of diacetyl, in *Lactococcus lactis,* is stimulated in acidic conditions and seems to be produced to control intracellular pH [85]. Papagianni [86] has examined several approaches that have been adopted to improve the production of diacetyl. In aerobic conditions, the pyruvate pathway is shifted to the synthesis of α-acetolactate, the reaction is catalyzed by two enzymes: α-acetolactate synthase (ALS) or acetohydroxy acid synthase (ILVBN). Acetolactate can be either converted into diacetyl by an oxidative reaction or into acetoin by decarboxylation with the enzyme α-acetolactate decarboxylase (ALDB). Genetic engineering has attempted to inactivate the gene that expresses ALDB and to overexpress the ALS and ILVBN genes, with little success. In contrast, the overproduction of NADH oxidase (NOX) in *Lactococcus lactis*, in addition to the inactivation of ALDB gene, has been shown to be successful in driving the metabolism of pyruvate in the production of diacetyl, rather than lactate [87]. In this way, *L. lactis* has increases diacetyl production at the same time as it reduces lactate production [88]. 

In this context, many efforts have been aimed at the construction through metabolic engineering of recombinant strains to enhance flavor production, as well as to the screening of LAB collections, in particular *Lactobacillus* spp., to determine their metabolic potential for the synthesis of flavor compounds and create a better definition of acetoin production through transcriptional activation [89,90,91].

In recent studies, lactic acid fermentation was selected as a process for enhancing the flavor profile of fruit juice. Although most volatile compounds occur naturally in plants, they can be synthesized by microorganisms as secondary metabolites. Chen et al. [92] observed a positive modification on the aromatic profile of apple juice fermented with different LAB strains (belonging to the genus *Lactobacillus*), particularly an increase of alcohols like 2-ethylhexanol and ethyl acetate with floral and fruity notes. The same results were recorded in other fruit juices such as pomegranate [93], where the fermented juice, with *L. plantarum,* had a more intense fruity note that can result from an increase in alcohols, ketones, and terpenes; or in elderberry juice [70], where LAB increased the volatile compounds typical of elderberr, like β-damascenone and various alcohols such as hexanol, 3-hexen-1-ol (Z), 2-hexen-1-ol (E), ethanol, 2-phenylmethanol, 2-phenylethanol, isoamyl alcohol, hexanol, 3-hexen-1-ol (Z), and 2-hexen-1-ol (E). During the fermentation of barley malt wort beverages [94] with different LAB strains, a higher aroma yield and fruity flavor was recorded, due to the increase of some compounds like β-damascenone, furaneol, 2-phenylethanol, and ethyl 2-methylbutanoate.

The activation of the metabolic pathways that lead to the formation of certain aromatic compounds is, however, closely related to the strain and the substrates used for fermentation. Thanks to the unique portfolio of enzymes that LAB possess, they activate different metabolisms, such as the catabolism of aldehydes, the synthesis and hydrolysis of esters, the degradation of phenolic acids, lipolysis, proteolysis, and peptidolysis [95].

Despite the number of studies on lactic acid fermentation showing an improvement in the sensory qualities in fermented products, the use of LAB for the production of aromas from waste and byproducts has been rarely studied [79,80]. LAB can grow on many different substrates, among which are ligno-cellulosic byproducts, agri-food, and municipal solid wastes [96,97].

Almost all LAB are not able to directly ferment complex polysaccharides like starch or hemicellulose, and therefore hydrolysis pretreatments (with related costs) are necessary, together with the addition of amino acids, nucleotides, and vitamins [78]. For the direct exploitation of these economic feedstocks, some strategies have been implemented, including the co-cultivation of LAB with native cellulolytic microorganisms and the modification of the gene pool by inserting hydrolytic genes for saccharification [98]. Escamilla Hurtado et al. [99] reported that the production of diacetyl with *Pediococcus pentosaceus* and *Lactobacillus acidophilus* increases on starchy substrates according to different parameters. 

Moreover, the use of Kefir (granules containing lactic acid and acetic bacteria and fermenting yeasts) in SSF on food industry wastes showed significant production of ε-pinene (Table 1), with an estimated yield in biorefineries of 4 Kg per ton of treated substrate [50].

## 5. Conclusions

Recent years have witnessed a rapid evolution of different methods of natural flavor and fragrance chemical production through biotechnological routes. The use of microbial cultures offers several advantages over traditional methodologies, such as the possibility to label flavors as “natural,” thus making them more attractive for consumption, with market acceptability. Agri-food waste exploitation can be of great help, often offering excellent substrates for microbial growth and enhancing waste recovery and valorization at the same time. High operating costs are among the main issues to solve for the implementation of these production systems. The employment of LAB offers the possibility of adding value to agri-food waste by producing natural flavors. Their use offers advantages compared to other microorganisms, such as GRAS status and good adaptability to different carbon sources and environmental stress, with the disadvantages being that they often need pretreatment to make sugar available and nutrient supplementations. FWs used as a substrate can overpass the last issue. Moreover, the techniques of genetic modification in LAB are being rapidly developed thanks to the completion of genome sequencing and the wide availability of handling techniques. In light of the wide applicability of LAB fermentations, few studies have addressed the efficiency of this approach for the synthesis of aromatic compounds using agri-food waste as a substrate.

Undoubtedly, a careful assessment of production and downstream costs will be required to guarantee the economy of the design process. SSF technology has yet not been fully implemented at the industrial scale because of the lack of easily scalable reactors able to successfully overcome the problems with heterogeneity and sterility. Consequently, further targeted studies are needed to assess the most effective methods for the extraction and separation of flavor compounds at the industrial scale.

## Figures and Tables

**Table 1:** Aroma production by SSF of agri-food wastes/byproducts, using molds and yeasts.

	Agri-Food Waste	Pretreatment(s)	Aroma	Reference
Mold				
*Ceratocystis fimbriata*	Citrus pulp + 25% sugarcane molasses (+50% soya bran)	Drying, milling, sieving	Fruity aroma	[56]
	Coffee husks (+glucose)	Milling, steam treatment	Pineapple aroma (acetaldehyde, ethanol, isopropanol, ethyl acetate)	[49]
	Coffee husks	Drying, milling, sieving, sterilization	Fruity flavor	[57]
	Cassava bagasse, apple pomace, amaranth, soybean	Drying, milling, sieving, sterilization	Fruity aroma (+ amaranth and + banana aroma)	[52]
*Rhizopus oryzae*	Wheat bran, cassava bagasse, sugarcane bagasse	Milling, sieving, sterilization. For sugar cane bagasse: preliminary washing	Fruity aroma (strong banana aroma)	[58]
	Cassava bagasse, apple pomace, soybean, amaranth, soybean oil	Grinding, drying, sterilization	Acetaldehyde, Ethanol, 1-Propanol, Ethyl acetate, Ethyl propionate, 3-Methyl butanol	[59]
*Trichoderma viride*	Sugarcane bagasse	N.d.	Coconut aroma, 6-pentyl-α-pyrone	[60]
		Drying, milling	Coconut aroma, 6-pentyl-α-pyrone, from δ-Octalactone to Dodecalactone	[61]
*Trichoderma harzianum*	Sugarcane bagasse	Drying, milling	6-Pentyl-α-pyrone	[62,63]
*Kluyveromyces marxianus*	Apple pomace, cassava bagasse, sugar cane bagasse, sunflower seeds, giant palm	Drying, milling, sieving, sterilization	Ethanol, ethyl acetate	[64]
	Sugarcane bagasse + sugar beet molasses	Drying, milling, pH adjustment	Fruity aroma (43% alcohol, 35% esters)	[54]
		Drying, milling, pH adjustment	Fruity aroma	[55]
*Aspergilius niger, Penicilium cinnabarium*	Rice brain oil residue (+ferulic acid)	Water-ethanl extraction, pH adjustment, filter sterilization	Vanillin	[65]
*Hanseniaspora velbyensis and uvarum, Saccharomyces cerevisiae*	Apple peels	Drying, homogenization	132 volatile compounds	[66]
**Yeasts**				
*Pichia kudriavzevii*	Sugarcane bagasse + l-phenylalanine	Drying, milling, pH adjustment	Rose aroma	[67]
*Saccharomyces cerevisiae*	Citrus peels	Slicing, grinding	Isoamylacetate, ethyl dodecanoate, ethyl decanoate, ethyl hexanoate	[68]
*Yarrowia lipolytica* (engineered)	Fatty feedstock	N.d.	Coconut like flavor (γ-dodecalactone, ẟ-decalactone)	[69]
Kefir (symbiotic yeasts and bacteria)	Food industrial wastes (cheese whey, molasses, brewer’s spent grains, malt spent rootlets, orange and potato pulp)	Blending	Ɛ-pinene	[50]

## Data Availability

Not applicable.
